# Royal Infirmities

**Published:** 1881-03

**Authors:** 


### ROYAL INFIRMITIES.

In the days of Shakespeare's King Henry, it was said that "uneasy lies the head that wears a crown", but some prosaic fellow has recently informed us that it is a mistake to think that a sovereign goes to bed with his crown on. He simply takes it off, and hangs it on the back of a chair with his vest and the rest of his clothing. And just as rulers behave in this respect as other mortals would, so are they also subject to infirmities and afflictions like unto ourselves. It is well known that Prince Leopold, by some special providence, was born into this world having a skin of only two layers, instead of the three vouchsafed to most men. This peculiarity not only renders him an object of special solicitude during his lifetime, but, after his death, if his highness could be preserved in alcohol, properly labeled, and placed with the rest of the "pickles" in the Museum of the Royal College of Surgeons, that would indeed be a most rare and interesting specimen.

It was a favorite myth in earlier times that such personages sprung into existence in some inexplicable fashion, as did Minerva from the head of Jupiter. If it was not the doctor, nor a stork—as the German children believe—nor some other magician, who brought the baby, there was at least an element of the mysterious in his arrival. But unfortunately these fond delusions too, have vanished. A modern Prince is of little account unless his advent has been heralded abroad some months previously and other evidence produced, to show that he, like the rest of his class, has been produced in the ordinary fashion. This is perhaps not to be wondered at when important questions of State depend upon the succession.

The American reader of foreign journals is often surprised however, by personalities which are apt to shock his sense of propriety. For example, a recent number of *La France Medicale* contains an article on "The fourth pregnancy of the duchess de Berry and the birth of the duke de Bordeaux." It may be of intense scientific interest to ascertain the exact antero-posterior diameter of such a royal pelvis, or other minutiæ relating to a confinement, but in this country at least, ladies are allowed a certain degree of privacy in these transactions, which may well be a source of envy for their sisters abroad.

